# Acceptance of provider–initiated testing and counseling for HIV infection by caregivers in a tertiary health institution in Abuja, Nigeria: a cross sectional study

**DOI:** 10.11604/pamj.2016.24.245.9057

**Published:** 2016-07-15

**Authors:** Adaora Adeline Okechukwu, Eno Ekop, Chinwendu Daniel Ndukwe, Kudirat Eyinade Olateju

**Affiliations:** 1 Department of Paediatrics, University of Abuja Teaching Hospital, Gwagwalada, Abuja; 2 Department of Programs Coordination, National Agency for the Control of AIDS, Abuja

**Keywords:** Provider initiated voluntary counseling and testing, HIV, uptake, children

## Abstract

**Introduction:**

Less than 10% of HIV positive children are enrolled into antiretroviral treatment program in the country. Provider-initiated testing and counseling was introduced to increasing uptake of HIV testing. The aim of this study is to determine the acceptability and factors undermining the acceptance of this laudable initiative by parents/caregivers of children attending paediatric out patient clinical services in our health institution.

**Methods:**

A cross sectional study of children aged 18 months to 18 years and their parents/caregivers attending paediatric outpatient clinic of the hospital was undertaken for the above objectives.

**Results:**

There were statistically more female parents/caregivers (82.5%, p=0.00), more male patients (52.9 %, p= 0.02), and 11.9% adolescents in this study. While 91.7% of parents/caregivers admitted not having knowledge of provider-initiated testing and counseling, 95.6% knew what HIV was. Acceptance of the program was high (98.7%), majority (89.7%) wanting to know the HIV status of their children/wards. Non-acceptance was small (1.2%), there main reason being prior knowledge of their HIV status. Prevalence of HIV among tested children was 1.7%. There was a strong relationship between having willingness to test for HIV and many of the study variables with religion of the parents/caregivers having the strongest relationship [OR: 13.94, (CI 1.82, 55.34)], and tribe having list association, [OR: 3.60, (CI 1.85, 17.14)].

**Conclusion:**

There was general wiliness to accept HIV test for children by their parents/caregiver in this study, and HIV prevalence in children is on a downward trend; its sustenance to be continued and adolescent clinics need to be created.

## Introduction

Thirty-four million people worldwide are estimated to be affected with the Human Immunodeficiency virus (HIV) with sub-Sahara Africa alone accounting for 72% of new infections [1]. Nigeria ranks third behind South Africa and India in global burden of this disease with over 3.3 million of its population living with HIV, out of which 360,000 are children below the age of 15 years [2, 3].

Despite the huge burden of disease in the country and in the sub-region, over 80% of HIV infected adults do not know their status [4, 5]. This figure is even worse in children where very low uptake of voluntary counseling and testing (VCT) has also been recorded. [6–9]. Akhigbe et al. [9] noted that of 1,490 people who had accessed VCT services at a primary health care center in Nigeria over a 3 year period, only 3.5% were children less than 14 years of age. Many patients get to know their HIV status at a late stage in the disease, after having made many visits to health facilities during which there would have been many missed opportunities for counseling and testing these patients [10, 11].

Provider-initiated testing and counseling (PITC) was introduced by the World Health Organisation (WHO) in 2007 with the aim of increasing the uptake of HIV testing, improving access to health care services for people living with HIV and creating new opportunities for HIV presentation [4]. Early diagnosis of HIV in children is very important because the disease has a more rapid course when compared to adults; the signs and symptoms are not specific and mimic most childhood illnesses [12]. One-quarter of HIV infected children will die before the age of 1 year while most die before their fifth birthday if they do not have access to care [12]. PITC “refers to HIV testing and counseling recommended by health care providers to persons attending health care facilities as a standard component of medical care [4]. With PITC, the patient presenting to a health care facility is offered HIV test by the health care provider irrespective of his or her presenting complaints. This differs from the conventional client-initiated testing and counseling (also known as VCT) where the patient actively seeks out the health care facility and requests to be tested [13]. PITC aims to diagnose early the unrecognized or unsuspected HIV infected persons attending health facility, which can take the form of “opt-in” or “opt-out” approach [4]. In the opt-in approach, the patient must accept to have the HIV test done after receiving pre-test counseling. The informed consent is obtained in a manner similar to that required for special investigations or interventions in clinical settings for example, performing a liver biopsy. In the opt-out approach, the patient may refuse to have the test performed if they chose not to accept the offer after receiving the pre-test counseling. The test is offered in a manner similar to that for common clinical investigations like complete blood count and non- invasive investigations [4].

The recommendations for implementing PITC vary according to the burden of HIV in that country: low-level HIV epidemics, concentrated HIV epidemics and generalized HIV epidemics [4]. Nigeria falls under the generalized HIV epidemics category. In this category, HIV is firmly established in the general population with HIV prevalence of pregnant women consistently over 1% [4]. WHO recommends that in such a country, PITC should be offered to all HIV-exposed infants or infants born to HIV exposed women as a routine component of follow-up care for these children. PITC should also be offered to children who present with suboptimal growth or malnutrition, and malnourished children who are not responding to appropriate therapy. Thirdly, that adolescent health services be made a priority in the implementation of PITC [4]. PITC is feasible and can be used to identify many undiagnosed infected children who are at increased risk for mortality [14]. PITC has been reported to increase the uptake of HIV testing. A study reported an increase ranging from 9.9% - 65.6%, and 5.5%-78% after the introduction of PITC in antenatal clinics [15]. It has been implemented in several clinical settings in many developed and developing countries like Botswana [16], Uganda [17], Kenya [18], Haiti [19], Rwanda [20], Malawi [7], Zambia [8], Uganda [14], Canada, USA and the United Kingdom [4]. A study in Uganda among 1221 patients aged 15 to 49, reported a three - fold increase tested per practitioner in the number of patients accepting to be tested for HIV [21]. However, there are few studies on PITC in West Africa, including Nigeria and even fewer studies done using the paediatric age group. Most studies available are on adult populations, and mainly using the VCT approach. This study is aimed at determining the prevalence of HIV infection among children presenting at the paediatric out-patient clinics, and to identify socio-demographic variables associated with PITC acceptance and non-acceptance at the unit of the hospital using the opt-out PITC approach.

## Methods

A cross sectional, observational, hospital-based study was carried out at the out-patient clinic of paediatric department of the University of Abuja Teaching Hospital (UATH), Gwagwalada, Abuja, Nigeria from 5^th^Febuary to 12^th^ March 2015. UATH is a 350 bedded tertiary health care center located in Gwagwalada Area Council, one of the six area councils in the Federal Capital Territory (FCT), Abuja, Nigeria. The hospital serves its host community, Gwagwalada, the other parts of FCT include Nasarawa, Kogi, Kaduna and Niger States. Paeditric out-patient clinic (POPC) is a unit in the department that provides out-patient clinical services to all children aged 1 day to 18 years in the hospital. It is opened to clinical services from Mondays to Fridays,8am to 4pm, excluding public holidays. The clinic includes a general out-patient clinic service area, and eight (8)specialized clinics (cardiology, nephrology, haematology, neonatology, neurology, oncology, infectious disease, gastroenterology, and endocrinology). The children are attended to by resident doctors undergoing training in paediatrics, paediatric consultants, a medical officer, nurses, pharmacists, record clerks and other support staff. There is also a separate paediatric out-patient special treatment clinic (POSTC) services for HIV infected children and exposed babies within the health facility that wasn’t included as part of the study.

### Study procedure

At the waiting hall of POPC, general pretest counseling was offered to the caregivers/ parents of the patients and adolescents by a trained paediatric VCT counselor in accordance with the Nigerian guidelines on paediatric HIV and AIDS Treatment Care [12]. The counseling include reasons for offering the test, the benefits of testing early, confidentiality for the test result, prompt enrollment into the HIV care and support programme at the POSTC in the health facility if the result turns out positive. They were also informed that the test would be at no additional financial cost to them. The children and adolescents, who meet the inclusion criteria, were then offered questionnaire to fill. The non-literate ones will have their questionnaires filled for them by the research assistant. Inclusion criteria include: parents/caregivers of patients or adolescents themselves who presented to the POPC for clinical services and aged between 18 months to 18 years, consent from the caregivers, and ascent for adolescents (9 to 18 years). Exclusion criteria: refusal to give consent by caregivers, refusal to assent by adolescents, orphaned, abandoned, mentally or intellectually disabled children. Sample size of 720 was calculated using formulae by Araoye [22], and recruitment of parents/caregivers and adolescents into the study was continue until the required sample size was met. After filling of the questionnaire, the caregiver/parents or adolescents themselves offered to have HIV test done for his or her child/ ward or him/herself and given the opportunity to “opt-out” if so desired, they were informed that refusal to have the test done will not affect the care the child will receive in the hospital. Signed or thumb printed consent was then be obtained for participating in the study before having the HIV test done. Parents/caregivers or adolescents who refuse to participate were noted and the reason for the non-participation documented.

HIV testing was done in series using rapid diagnosing test kits in line with the Paediatric National Guidelines by a trained resident doctor in paediatrics on HIV testing. All the paediatric doctors in the department were trained and retrained on HIV testing method by a laboratory scientist in the hospital. The Determine™ test kit was used for the initial test. If the test turns out negative, no further testing was carried out, and the child was reported as negative for HIV. If the test was positive, a second blood sample was tested for a second test using a different test kit, Uni-gold™. If the second test was positive, then the patient is reported as positive for HIV. If the second test was negative, then a tie-breaker test was done with yet a new blood sample using a third different test kit, the statpak™. If the statpak kit test was positive, then the patient was reported as positive for HIV, but if negative, the patient is reported as negative. Post-test counseling was done for each participant individually regardless of whether the HIV test result was positive or negative in a quiet, secluded place in accordance with the National Guidelines [12]. All positive participants were referred to the POSTC in the hospital for enrollment, treatment and care.

A structured questionnaire developed by the research team was self-administered to the literate caregivers, while the non-literate ones were filled by research assistant via a face-to-face interview. These included questions on socio-demography of the participant and caregiver (name, age , sex, tribe , religion, occupation, educational status), relationship of caregiver to child, reason for visit to hospital, knowledge of HIV, PITC, history of previous HIV test, reason(s) for acceptance or non-acceptance of PITC and reason(s) for not having been previously tested.

Ethics approval was obtained from the Health Research and Ethics Committee of the hospital before the commencement of the study, and informed consent obtained from the caregivers/parents. The principles of research ethics were highly maintained. There was no conflict of interest.

Data analysis was done using SPSS version 20. Frequency and chi square was used for categorical variables. Continuous variables was also be analyzed. A probability of of 5% was regarded as significant. Analysis of social class was based on the classification by Olusanya [**23**].

## Results

Table 1 depicts the characteristics of the study population. There were statistically more male patients 396/748 (52.9%, p=0.02), more female parents/caregivers 617 (82.5%, p=0.00), and 11.9% adolescent patients. The mean age, and body weight of male and female patients were not statistically different (43.6 ±2.4 Vs 45.3 ±3.2 months) and (15.2 ±0.6 Vs 16.8±0.7 kg), p values were 0.99 and 0.46 respectively, and majority of the patients 524 (70.1%) seeking clinical services in the POPC of the hospital were children less than five years of age. There were statistically significant more female caregivers (82.5%), p=0.00, more mother-primary-caregivers (81.6%, p=0.00), more Christians (71.4%, p=0.00), and more married couples (98.4%, p=0.00) among the caregivers/parents interviewed. There were also more caregivers with tertiary level of education (52.0%), and more from upper socio-economic class (43.3%).

**Table 1:** Characteristics of the study population

Variables	Male (%)	Female (%)	Total (%)	P value
sex	396(52.9)	351 (46.9)	748 (99.8)	0.02
Age (months)	43.6±2.4	45.3 ±3.2	44.4±1.9	0.09
Weight (kg)	15.2±0.6	16.8± 0.7	16.0 ±1.6	0.46
**For Caregivers**				
Sex	125(16.7)	617(82.5)	748(99.2)	0.00
Age (years)	9.6±0.7	32.3±0.3	3.9±0.3	0.00
**Educational Level**				
Primary	7(11.7)	53(88.3)	60(18.0)	0.00
Secondary	41(15.4)	21(83.1)	226(35.6)	0.00
Tertiary	72(18.5)	315(80.9)	389(52.0)	0.00
No education	5(17.9)	1(82.1)	28(3.7)	0.00
**Socio-Economic Status**				
Upper	75(23.1)	247(76.2)	324 (43.3)	0.00
Middle	32(14.0)	195 (85.5)	28 (30.5)	0.00
Lower	18(9.2)	175 (89.3)	196 (26.2)	0.00
**Relationship with**				
the Patients				
Aunty	0.0	2(100)	2(0.3)	
Father	119(15.9)	0.0	119(15.9)	
Mother	0.00	610(81.6)	610(81.6)	
Guardian	1(6.25)	15(93.8)	16(2.1)	
**Religion**				
Christian		85(15.9)		444(81.6)
Muslim		40(18.7)		173(80.8)
**Marital Status**				
Married	123(16.7)	608(82.6)	736 98.4)	
Single	2(16.7)	9(75.0)	12(1.6)	

Table 2 showed the knowledge of PITC by parents/caregivers/adolescents. Majority of the parents/caregivers/adolescents686 (91.7%) interviewed have not heard about PITC before, however, 715 (95.6%) did indicate of having knowledge of what HIV was, and 738 (98.7%) accepted and gave consent to have HIV test carried out for their children/wards/ themselves in cases of adolescents, only 9 (1.2%) decline to the test. All 748 (100%) admitted they have not tested their children/ wards/themselves for HIV before. The characteristics of the parents/caregivers who decline from having HIV test done for their children/wards include: 6 (66.7%) were males, 5 (55.6%) does not have any formal education, 7 (77.8%) were from Islamic religion and 3 (33.3%) were adolescents. Reasons for non-acceptance by few (1.2%) include: I have already known by HIV status (55.6%), I am in a hurry for the test, and i don’t want my child/ward to cry from any needle prick (44.4%), the need to get permission from their husbands (33.3%). An interesting aspect of the test was that 671(89.7%) of the parents/caregivers who showed willingness to test their child for HIV did so because they wanted to know their status, while (5.2%) believed that there child/ward will be negative.

**Table 2:** What caregiver Knowledge about PITC

Have you heard about HIV before	Total (%)
Yes	715(95.6)
No	33(4.1)
**Have you heard about PITC**	
Yes	54(7.2)
No	686 (91.7)
**Has your child been tested for HIV before**	
Yes	0(0.0)
No	748(100.0)
**What was your reason for not testing your child before**	
I don’t know a child can have HIV	132(17.6)
I don’t know where to go for the test	2(0.3)
I have never been asked to perform the test	610(81.6)
No reason	4(0.5)
**Will you like your child to be tested**	
Yes	738(98.7)
No	9(1.2)
**What was your reason for not consenting for the test for your child**	
My child can never have HIV	0(0.0)
I need to get permission from husband	3(33.3)
I have tested the child before	0(00.0)
Child is very ill and needle prick will make him/her to cry	3(33.3)
I have already known my status	5(55.6)
I am in a hurry	4(44.4)
**What was your reason for accepting/consenting to test your child**	
I have already know my child status	0(0.0)
I believe my child will be negative	39(5.2)
I will like to know	671(89.7)
It will tell me my status	17(2.3)
No particular reason	18(2.4)

Table 3 showed the HIV test results of the patients. While 13(1.7%) of the patients tested were positive, 7 (53.8%) male, and 6 (46.2%) were females, majority of the test results 734 (98.1%) turned out to be negative. All the positive results were children <5 years of age, none was adolescent.

**Table 3:** HIV test results

Age (Years)	Negative (%)	Positive (%)	Total (%)
0 – 4.99	511 (97.5)	13 (1.7)	524 (70.1)
5 – 9.99	135 (100)	0 (0)	135 (18.1)
10 – 14.99	75 (100)	0 (0)	75 (10.0)
15 – ≤18	14 (100)	0 (0)	14 (1.9)
Total	734 (98.1)	13 (1.7)	748 (100)

Table 4 showed the relationship between various study variables of parents/caregivers and patients and willingness to test for HIV. There was a strong relationship between having willingness to test for HIV and many of the study variables with religion of the parents/caregivers having the strongest relationship [OR: 13.94, (CI 1.82,55.34)], and tribe having list association, [OR: 3.60, (CI 1.85, 17.14)].

**Table 4:** Relationship between willingness to test for HIV and study variable

Variable	OR	CI
Age of patients	4.692	1.717-13.325
Age of care givers	6.372	2.176-19.667
Tribe of caregiver	3.603	1.850-17.146
Sex of patients	6.766	1.604-32.06
Sex of care givers	5.530	2.025-15.694
Marital status of caregiver	6.614	2.424-18.758
Education level of caregiver	5.030	1.190-23.878
Occupation	8.316	1.976-92.06
Family size	4.441	1.628-43.829
Relationship with patients	5.466	2.002-15.514
Religion of caregiver	13.939	1.823-55.34

## Discussion

Acceptance of PITC by parents/caregivers/adolescents in POPC of our hospital was high 98.7%. This was comparable to 98.2% and 89.9% among admitted children in Zambia [24] and Uganda [17] and 99% among out-patient attendees in Rwanda [20], and Cameroun [25]. The finding was also comparable to 99.4% earlier reported from a multi-center study in Nigeria by Nguavese et al in 2014 [26]. It was however contrast to 54.2% from consented caregivers to HIV testing from six primary health care clinics in Zimbabwe [27]. The high acceptance of PITC by parents/caregivers of children attending POPC in this study might be as a result of high knowledge of HIV among them. Other possible reason being that greater proportion (89.7%)of parents/caregivers wanted to know the HIV status of their children/wards. Similar reason was given for high acceptance of PITC by parents/caregivers of clinic attendees in Rwanda [20], most of whom wanted to know the HIV status of their children, and for the fact that the HIV testing was conducted by the health workers themselves. Globally, Nigeria has the highest annual number of children acquiring HIV [28], and PITC has a key strategy in increasing paediatric HIV testing and enrollment into treatment and care. This strategy has been reported to have increased HIV testing uptake in many healthcare service centers across the globe [20, 21, 24–27, 29], from 40.8% to 98.2%in Zambia[8], and 3.3% to 76.0% in Zimbabwe [29]. It is also being implemented in several clinical settings in many developed and developing countries [4]. This high level of acceptance of PITC in this study is a positive step in scaling up of paediatric HIV testing and enrollment of positive children in our health institution and elsewhere, as only <10% of positive children needing ART in the country has been reported to have enrolled for treatment and care [26, 28]. Its implementation in all the key paediatric service areas in the hospital and elsewhere will further help in identifying missed positive children for early treatment, care and support, as this will in no immeasurable way help in the reduction of morbidity and mortality from HIV infection in children.

Reasons for non-acceptance of PITC in many developing countries are multifactorial, ranging from individual/ community misconceptions, to health care facility inadequacies, and poor national legal framework. There is generally perceived lack of importance of HIV testing for children by their parents/caregivers, their health care providers, their policy-makers, and the children themselves [30, 31]. There are common misconceptions; that HIV testing is required for only symptomatic children, and that peri-natally infected children do not survive into late childhood [30, 31]. Lack of knowledge of serious consequences of untreated infection in apparently asymptomatic or mildly symptomatic children; and that recurrent subtle symptoms like skin infections, poor weight gain, and school failure are often not considered suggestive of HIV are also common misconceptions [30, 31]. Disclosure and stigma issues are also contributory to the general non-acceptance of PITC in most developing countries. The general believing that disclosure of parent status and the child´s knowledge of his/her HIV status will cause stress and exacerbate his/her disease is a common phenomenon [30, 31]. Hence parents//caregivers will want to protect their children and themselves from discrimination within the family and community by not wanting to know their HIV status, or not accepting PITC. Facility-level barriers include lack of child-friendly services, negative health workers attitude, insufficient staff and equipment, non-availability of HIV test kits, and prohibitive cost of travel distances for the test [30]. HIV testing non-acceptance was quite` low (1.2%) in this study. This was also reported from other studies, 1.8%, 1.0% and 0.9% from Zambia [24], Rwanda [20], and Cameroun [25]. It was however contrast to 45.8% obtained by Kranze et al [29] from Zimbabwe. In the Zimbabwe study, the main reasons for the high number of non-consenting to HIV testing were perceived unsuitability of the accompanying guardian to provide consent for HIV testing on behalf of the child and lack of availability of staff or HIV testing kits. They also did mention hat asymptomatic children, or older children, or those attending the clinic with a male or a younger guardian had significantly lower odds of consenting to HIV testing. This was in contrast to the reasons for non-consenting of HIV test in this study where parent/caregiver who did not consent mention their prior knowledge of their HIV status as their main reason for not consenting, while some said they were in a hurry to wait for the test, and the women among them needed to get permission from their husbands before the test.

The much lower prevalence of HIV (1.7%)as documented in this study differs from 11.9% earlier reported in same unit of the hospital in 2011 [32], and 8.4% in a multicenter study in the country in 2014 [26]. This downward trend in HIV infection is not only reflected in the national sero-prevalence rate, but also in acquisition of new infections, a trend from 5.8% in 2001 to 3.4% in 2012, and new infection from 338,423 in 2005 to 176,701 in 2015(reflecting 50% drop) [28]. This positive step could be as a result of improved funding of HIV programs by the Federal Government of Nigeria, the international partners, relevant agencies, and non-governmental organizations. Extensive awareness of HIV by Nigerians, and signing into law of the Anti-Discrimination Bill in 2014, which gave hope to those living with the disease that they would not be stigmatized when other people are aware of their status could also be a contributory factor to the observed downward trend of HIV infection. The other possible reason for the low prevalence of HIV in this study might be due to exclusion of <18 months of age from the study. This group of children requires DNA PCR test for confirmation of HIV positivity, and with this exclusion more positive children may have been missed.

Adolescence is a transitory stage of development between childhood and adulthood corresponding to the period between the ages of 10 and 19 years [33]. It is a critical phase in the configuration process of manas habits and behaviors which will affect the life of the individual are cultivated at this time. There is no question about the fact that adolescents are susceptible to psycho-substance abuses, sexually transmitted diseases and other related ills in the society. This group of individuals often lacks basic reproductive health information, knowledge, and access to affordable confidential health services for reproductive health. It is therefore of utmost importance that they have guidance and the required health services at their disposal to help them make positive lifestyle choices to prevent ill health in the future. Despite the fact that the burden of adolescent health challenges are increasing in sub-Saharan African countries, there is currently low system capacity to address these issues with effective interventions in this area. The capacity of relevant professionals to address adolescent health issues through effective programming is poor throughout Africa. Traditionally, adolescents have had limited access to sexual health services in developing countries [34], although this situation is changing gradually.

In this present study 11.9% of patients assessing clinical services in POPC of the hospital were adolescents. This raises question(s) as to where most of this young individuals in our environment get their much needed health needs. Establishment of adolescent well clinics or sexual reproductive health unit to cater for their health needs will help to bridge this dangerous gap. Providing a safe and confidential center for young people is urgently needed as experience has shown that they would rather patronize quacks instead of the hospital as these quacks are perceived as “confidential though not safe”, while the hospitals are ‘safe’ but not ‘confidential’. It is therefore important to create a safe and confidential place for these young people.

## Conclusion

There was general wiliness to accept HIV test for children by their parents/caregiver in this study and HIV prevalence in children is on a downward trend; its sustenance to be continued and adolescent clinics need to be created.

### What is known about this topic

- Despite the huge burden of HIV infection in the sub-region, over 80% of HIV infected adults and even more in children do not know their status;
- PITC was introduced by the WHO to increase the uptake of HIV testing, improve access to health care services for people living with HIV and create new opportunities for HIV presentation.

### What this study adds

- PITC initiative was well accepted by parents/caregivers;
- Many children were tested for HIV by this method instead of the conventional client initiated;
- HIV in children in this environment is on a downward trend.
